# Investigating microbial activities of electrode-associated microorganisms in real-time

**DOI:** 10.3389/fmicb.2014.00663

**Published:** 2014-11-28

**Authors:** Sanja Aracic, Lucie Semenec, Ashley E. Franks

**Affiliations:** Applied and Environmental Microbiology Laboratory, Department of Microbiology, La Trobe University, Melbourne, VIC, Australia

**Keywords:** bioelectrochemical systems, electricigens, real-time gene expression, biofilms, *Geobacter*, electrodes

## Abstract

Electrode-associated microbial biofilms are essential to the function of bioelectrochemical systems (BESs). These systems exist in a number of different configurations but all rely on electroactive microorganisms utilizing an electrode as either an electron acceptor or an electron donor to catalyze biological processes. Investigations of the structure and function of electrode-associated biofilms are critical to further the understanding of how microbial communities are able to reduce and oxidize electrodes. The community structure of electrode-reducing biofilms is diverse and often dominated by *Geobacter* spp. whereas electrode-oxidizing biofilms are often dominated by other microorganisms. The application of a wide range of tools, such as high-throughput sequencing and metagenomic data analyses, provide insight into the structure and possible function of microbial communities on electrode surfaces. However, the development and application of techniques that monitor gene expression profiles in real-time are required for a more definite spatial and temporal understanding of the diversity and biological activities of these dynamic communities. This mini review summarizes the key gene expression techniques used in BESs research, which have led to a better understanding of population dynamics, cell–cell communication and molecule-surface interactions in mixed and pure BES communities.

## INTRODUCTION

Research into the field of electromicrobiology has grown exponentially over the last decade. Electroactive microorganisms can utilize electrodes either as electron acceptors (termed electricigens) or electron donors (termed electrotrophs). These organisms have a broad range of applications in bioremediation, biofuel production, energy production, and biosensing through the use of a variety of bioelectrochemical systems (BESs; Nevin et al., 2010; Rosenbaum and Franks, 2014). BESs can be divided into microbial fuel cells (MFCs), which produce a net current, and microbial electrolysis cells, which consume current providing reduction potential for the production of biofuels such as hydrogen or methane gas.

Microorganisms in BESs are often found in biofilms on the electrode surface and can comprise bacteria, yeast and archaea (Lovley, 2006). The composition of electrode-associated communities is dependent on a number of factors such as influent substrate, pH, oxygen concentration, and temperature (Logan and Regan, 2006; Wrighton et al., 2008; Patil et al., 2011; Lu et al., 2012). Electroactive microorganisms have been enriched on electrodes from a wide range of environmental inocula including rice paddy soil, aquatic sediments, sewage sludge, and compost (Zhang et al., 2010; Ringelberg et al., 2011; Nercessian et al., 2012). The use of BESs has allowed the study of extracellular electron transfer (EET) to and from insoluble electron acceptors and donors (Rosenbaum and Franks, 2014). However, our knowledge of these interactions is currently limited to a few well-studied bacteria, mostly species of *Geobacter* and *Shewanella*.

Initial studies within the electromicrobiology field focused on power production by electricigens utilizing MFCs for the generation of electrical current. While power outputs have increased exponentially, energy generation is still limited to powering small devices (Ieropoulos et al., 2013). A number of physical, chemical, and biological approaches have been employed to overcome limitations of these systems, however, a key limiting factor remains; a restricted understanding of microbial community dynamics and activities that occur within and between microorganisms in the presence of electrodes. Performance variables associated to BESs such as utilization of substrates, changes in pH, oxygen demand, and temperature have been monitored to understand the chemical limitations of these systems, to devise protocols for performance optimization (Jadhav and Ghangrekar, 2009; Patil et al., 2011). However, studies investigating microbial activities in real-time through gene expression are required for a complete understanding of BESs in order to overcome the biological limitations.

Numerous methods spanning a diverse range of scientific disciplines are employed in BESs research, some of which have been recently reviewed in detail (Zhi et al., 2014). The key techniques that have given insight into the structure and potential function of BES electrode-associated communities include high-throughput 16S rRNA gene sequencing and metagenomic sequencing. Although these methodologies have given a “snapshot” of the BES community at a particular time they do not allow temporal monitoring of gene expression. This review aims to summarize the key findings which give insights into the dynamic nature of electrode-associated communities using techniques that span the areas of microscopy, spectroscopy, electrochemistry, biochemistry, and molecular biology. Real-time gene expression studies are providing a more definite spatial and temporal understanding of the structural organization of the dynamic communities within BESs as well as their biological processes. Monitoring of the fluctuations in community structure and function will enable successful materialization of the powerful applications of BESs.

## PHYLOGENETIC DIVERSITY AND ACTIVITIES OF ANODOPHILIC COMMUNITIES

### MOLECULAR APPROACHES USED TO INVESTIGATE COMMUNITY STRUCTURE

**FIGURE 1:**
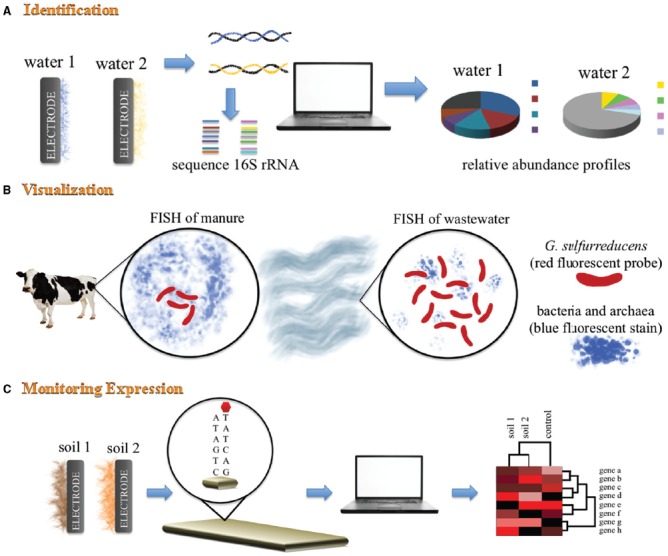
**Schematic representation of mixed BES community analyses. (A)** 16S rRNA gene sequencing analysis is used to identify dominant microorganisms found in electrode-associated communities. This fingerprinting technique provides an overview of the phylogenetic diversity within the electrode-associated communities. **(B)** Fluorescence *in situ* hybridization (FISH) using DAPi blue fluorescent stain for bacteria and archaea, and a species-specific probe for *G. sulfurreducens*. This technique allows identification, visualization and quantification of *G. sulfurreducens* within mixed BES communities from different environmental niches. **(C)** Geochip analysis of different metagenomic samples allows for specific genes of known function to be probed and identified.

The microbial diversity of electrode-associated communities in BESs is commonly investigated using denaturing gradient gel electrophoresis (DGGE), automated ribosomal intergenic spacer analysis, terminal restriction fragment length polymorphism, sequencing of the 16S rRNA gene via clone libraries or more recently, next-generation pyrosequencing (Figure 1). These fingerprinting techniques have been used to provide an overview of the composition of the electrode-associated communities from various soil and water environments and have often identified *Geobacter* as the dominant genus on the anode surface of BESs (Kan et al., 2011; Kiely et al., 2011b). The absence of *Geobacter* spp. from the electrode-associated community does not preclude power production, and ecological parameters such as the Shannon diversity index can be used as a stronger predictor of potential power output of a microbial community than taxonomic variables alone (Stratford et al., 2014). DGGE and 16S rRNA clone libraries provide a qualitative assessment of the relative abundance of microbial species in a mixed community, however, these studies are limited in depth of the diversity that they can detect. GeoChip and Phylochip have allowed examination of a larger diversity of the microbial community. For example, Gram-positive Firmicutes, particularly members of *Thermincola* and *Geobacillus*, have been identified as anode-reducing bacteria using high-density oligonucleotide microarray PhyloChip (Wrighton et al., 2008). This technique allows tracking of dominant organisms and minority populations and can uncover 35 times the diversity as compared to DGGE and clone libraries. GeoChip is similar to PhyloChip; although limited in number of taxon-specific oligonucleotide probes, it has the added benefit of having gene-specific probes allowing functional and structural analyses of stochastic processes within a BES (Zhou et al., 2013).

In-depth community analysis is now possible and can provide detailed “snapshots” of electrode-associated communities. For example, 3277 phylotypes representing 25 distinct phyla and 39 bacterial classes were identified in anodophilic communities using waste activated sludge (Lu et al., 2012). This community diversity allows for speculation about possible syntrophic interactions between phylogenetically diverse microbial populations through the utilization of organic matter. A recent study has proposed that *Sporomusa* converts methanol into acetate which is then utilized by *Geobacter* to generate electricity (Yamamuro et al., 2014). It can be expected that a higher diversity of anodophilic microorganisms will be identified in the near future with whole genome shotgun metagenomic analysis, and will provide a glimpse into the molecular potential within the anodophilic communities.

The stochastic and deterministic processes that influence the microbial community structure in anodophilic biofilms of BESs are not fully understood. Diverse microbial communities may be established in these biofilms despite deterministic factors being held constant such as influent substrate, temperature and applied voltage (Zhou et al., 2013). This highlights the need for tools that are able to track microorganisms and their interactions spatially and temporally in BESs due to the highly random nature of community dynamics in such systems.

### VISUALIZATION OF ANODOPHILIC COMMUNITIES

Bacterial populations within a BES are often visualized by scanning electron microscopy (SEM), a technique which requires dehydration of the electrode samples. Due to dehydration of SEM samples, dehydrated exopolysaccharides and pili may be mistaken for nanowires and identification of individual species in mixed communities is difficult. SEM images revealed that landfill BES anode biofilms contained predominantly microorganisms with bacilliform morphology and cell aggregates firmly attached to the anode surface with nanowire-like filamentous appendages (Puig et al., 2011). Thickness, architecture, spatial distribution and viability profile of microbial biofilms have to be determined in a hydrated state. These profiles are often determined using confocal scanning laser microscopy (CSLM), which requires staining the electrode samples with an appropriate DNA or cell wall stain. Using this technique, thicker biofilms were observed to correlate with increased current production (Pham et al., 2008) and Gram-positive and Gram-negative bacteria have also been observed to form microcolonies throughout an electroactive biofilm (Kim et al., 2006).

Phylogenetic-based techniques can be confirmed with fluorescence *in situ* hybridization (FISH) using genus-specific rRNA-targeted oligonucleotide probes to allow the spatial and temporal identification, visualization and quantification of targeted microorganisms in an electrode-associated community. Using this non-PCR based technique, *Geobacter sulfurreducens* was: identified to be present on anodes of sludge BES (Puig et al., 2011); found to be homogenously dispersed on the anode of a potato wastewater BES (Kiely et al., 2011a); and confirmed to contribute ∼60% of the anode biofilm community of a wastewater BES (initially quantitated using 16S rRNA clone libraries; Yates et al., 2012).

### MONITORING BIOLOGICAL ACTIVITIES OF ANODOPHILIC COMMUNITIES THROUGH GENE EXPRESSION STUDIES

Insight into the physiology of biofilm communities can be obtained through transcriptional profiling (An and Parsek, 2007). This high-throughput method has been used to link microorganisms and genes to specific physiological functions that occur within the electroactive community. Ishii et al. (2013) used metatranscriptomic analysis to characterize communities exposed to increasing and decreasing EET rates. Microorganisms that belong to the Desulfobulbaceae family were identified as predominant microorganisms (Ishii et al., 2013). These microorganisms have a number of EET-related genes encoding *c*-type cytochromes which are typically associated with *Geobacter*. The BES community harbored an abundance of sulfate reducers and methanogens that were consistently active within the community as analyzed by the mRNA/DNA ratio (metatranscriptomic versus metagenomic analysis). However, the sulfate reduction and methanogenesis pathways were not responsive to EET-related stimuli indicating that although present the genes were not expressed. Therefore there is a need to corroborate metagenomic analysis with metatranscriptomic analysis.

As cells in a biofilm are not all exposed to the same conditions, global analyses such as metagenomics and metatranscriptomics hide local microenvironments that occur within the biofilms. Future studies need to investigate the spatial and temporal effects of targeted gene-expression in response to environmental stimuli within mixed anodophilic biofilms. This requires detailed knowledge of the proteins involved and their specific roles in the EET pathways.

## MOLECULE-SURFACE INTERACTIONS IN PURE CULTURES OF MODEL ELECTRICIGENS, *Geobacter* AND *Shewanella*

### MUTAGENESIS STUDIES AND TRANSCRIPTIONAL ANALYSIS OF KEY GENES INVOLVED IN EXTRACELLULAR ELECTRON TRANSFER

The majority of electricigenic bacteria belong to the Proteobacteria phylum with a few belonging to the Acidobacteria, Bacteroidetes, and Firmicutes phyla (Logan, 2009; Ishii et al., 2012). Although a wide range of electricigens have been identified, the current understanding of EET mechanisms have come from pure cultures of *G. sulfurreducens* and *Shewanella oneidensis* (Lovley, 2012). Based on studies of these model organisms, three EET mechanisms have been proposed at the anode: electron shuttle mediated EET (SEET); pilin-mediated EET (PEET); and direct EET (DEET) which can also occur between different species of bacteria and is known as direct interspecies EET. SEET utilizes shuttles, which can either be produced by the microorganism or can be exogenous biotic or abiotic compounds, to transfer electrons to the anode. PEET occurs through pili via specific amino acid side chains in *G. sulfurreducens* (Vargas et al., 2013) or outer membrane protrusions in the case of *S. oneidensis* (Pirbadian et al., 2014). DEET commonly utilizes outer membrane cytochromes and requires cells to be in close proximity to the electrode. The monitoring of the redox state of outer membrane cytochromes is beneficial in determining community dynamics and the EET mechanisms employed.

**FIGURE 2:**
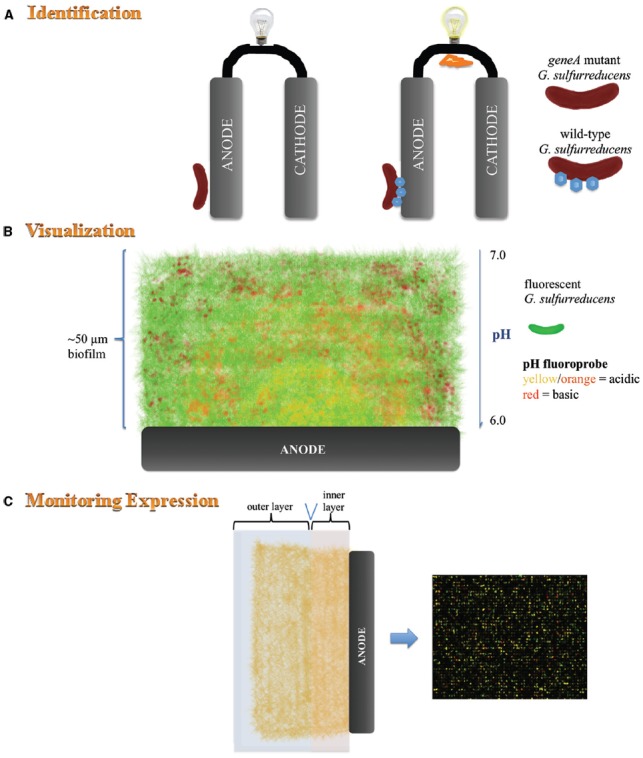
**Schematic representation of analyses conducted on pure cultures of *G. sulfurreducens*. (A)** Mutagenesis of gene/s encoding *c*-type cytochromes which play a key role in extracellular electron transfer to the anode results in inhibition of current production. **(B)** *In situ* monitoring of fluorescently labeled *G. sulfurreducens* (green fluorescence) within an anode biofilm stained with a pH-sensitive fluoroprobe. Confocal scanning laser microscopy reveals the thickness of the biofilm, proton gradient and location of active *G. sulfurreducens* throughout the biofilm. **(C)** Microarray analysis can be used together with other techniques such as microtoming to determine expression of genes at various distances from the anode.

The genome of *G. sulfurreducens* encodes more than 100 *c*-type cytochromes highlighting the complexity, flexibility and redundancy of its EET pathways (Methe et al., 2003). Genomic redundancy among *c*-type cytochromes is also observed in *S. oneidensis*, which encodes 42 putative *c*-type cytochromes (Meyer et al., 2004). In *G. sulfurreducens*, OmcZ and OmcS (depending on the BES) have been shown to be essential for optimal current production (Figure 2; Holmes et al., 2006; Nevin et al., 2009). Microarray and quantitative RT-PCR revealed that expression of *omcE* and *omcT* genes is higher in cells growing on electrodes than those growing with soluble electron acceptors providing support for their role in EET to anodes (Holmes et al., 2006). Interestingly, some *c*-type cytochromes such as OmcB are required for optimal EET to Fe(III) oxide and Fe(III) citrate but are not required for current production (Nevin et al., 2009). Numerous studies have made a link between expression of cytochromes and EET, however, the specific role of numerous *c*-type cytochromes in EET to the anode is still unclear.

A spatial examination using microtoming and microarray analysis of sectioned slices of a current-producing biofilm revealed that *G. sulfurreducens* cells at great distances from the anode, or within expected low-pH zones near the anode surface, are metabolically active and likely contribute to current production (Franks et al., 2010). Microarray analysis alone is not sufficient to provide a complete picture of the metabolic state of individual cells in a microbial biofilm (Franks, 2010). Real-time spatial gene expression analysis of *G. sulfurreducens* anode biofilms has been developed to overcome these limitations (Franks et al., 2012). Uniform expression of *pilA* and *omcZ* was detected throughout the biofilms using a gene encoding a short half-life fluorescent protein which was placed under the control of the *pilA* and *omcZ* promoters, respectively.

### VISUALIZATION OF EXTRACELLULAR ELECTRON TRANSFER PROTEINS AND CELL-ELECTRODE INTERACTIONS

Commonly used microscopic techniques to view anodophilic biofilms require the removal of the anode from the BES. However, real-time imaging of electrode-associated communities *in situ* provides continuous monitoring of development, from first attachment events to mature biofilms (Franks et al., 2009; McLean et al., 2010). A real-time, three-dimensional imaging approach was developed to visualize formation of a functioning anode biofilm community of *G. sulfurreducens* expressing mCherry fluorescent protein and its activity without disturbing the biofilm (Franks et al., 2009). This *in situ* technique allowed monitoring of the activity of BES anode biofilms spatially in real-time using CSLM imaging. The pH throughout different layers of the biofilm was measured by the addition of a pH-sensitive fluoroprobe. The pH decreased with increased proximity to the anode surface and from the exterior to the interior of the biofilm, demonstrating that proton accumulation was limiting current production. This drop in pH and decrease in current production correlated with reduced growth and activity of *G. sulfurreducens*. Strategies to facilitate the proton flux out of the biofilm or the use of electricigens which are tolerant to lower pH in BESs may improve current production.

Electron microscopy of immunogold labeled *c*-type cytochromes in *Geobacter* biofilms revealed that OmcZ is extracellular and is localized at the electrode surface (Inoue et al., 2010, 2011), OmcS is associated with pili (Leang et al., 2010) and that OmcB increases in abundance with distance from the anode (Stephen et al., 2014). This technique could be used to target other EET proteins for corroboration with *in situ* labeling methods such as FISH, and provide a quantitative understanding of how the conductive matrix changes to support EET in different regions of the biofilm.

It has recently been shown that *S. oneidensis* harbor extensions of plasma membrane (previously thought to be pili due in part to dehydration of SEM samples), containing cytochrome redox proteins (Pirbadian et al., 2014). The formation of these membranous extensions was observed using fluorescently stained membrane lipids and proteins *in vivo* via fluorescence microscopy, also identifying MtrC and OmcA cytochromes in the outer and periplasmic membrane extensions. To provide insight into the function of the membrane extensions, a fluorogenic dye activated upon interaction with reductases showed that production of these structures correlates to an increase in reductase activity, suggesting an increase in respiration and consequently SEET. The use of the SEET mechanism by *S. oneidensis* is further supported by *in situ* single-cell optical imaging using transparent nanoelectrodes as a platform which revealed that the current generated did not correlate with cell numbers on the electrode (Jiang et al., 2010). The finding supported the observation that low external resistance resulted in high current production per cell located within thin biofilms, while high external resistance resulted in low current production per cell located within a thick biofilm (McLean et al., 2010). The development of these biofilms was monitored using an optically accessible, continuous flow BES that enables real-time microscopic imaging of anodophilic populations as they develop from single attached cells to a mature biofilm.

### MONITORING THE FUNCTION OF PURE BES COMMUNITIES USING ELECTROCHEMICAL ANALYSES

Several electrochemical techniques have been used to investigate the performance and dynamics of pure and mixed BESs including: current–voltage polarization (determine the potential power output of the BES), electrochemical impedance spectroscopy and cyclic voltammetry. Electrochemical impedance spectroscopy can be used to identify electrochemical reactions occurring at electrodes, measure electron transfer resistance, determine the role of mediators in EET, and monitor biofilm formation in real-time (He and Mansfeld, 2009). Real-time *in vivo* monitoring of EET from an electricigen to the electrode can also be detected using cyclic voltammetry. This technique was used to demonstrate that *G. sulfurreducens* forms a conductive network of bound electron transfer mediators that most likely utilize OmcZ to transfer electrons through the biofilm and OmcB to transfer electrons across the electrode-biofilm interface; that pili are important in both reactions, that OmcS and OmcT are of secondary importance to the former, and that OmcE is not involved in EET during anode reduction (Richter et al., 2009).

During DEET between *G. sulfurreducens* cells and the anode, direct interaction of membrane cytochromes with the anode surface was shown using surface enhanced infrared absorption spectroscopy and subtractively normalized interfacial fourier transform infrared spectroscopy (Busalmen et al., 2008). Furthermore, *in situ* surface-enhanced resonance Raman spectroscopy revealed that DEET occurs via the heme groups of the outer membrane cytochromes (Millo et al., 2011). Microelectrodes probing *G. sulfurreducens* biofilms growing on electrodes using confocal Raman spectroscopy showed that outer regions of the biofilm were at low redox potential, and oxidized zones (containing reduced *c*-type cytochromes) were only detected in the inner portion (Stephen et al., 2014). Based on these observations it was proposed that extra DEET machinery is needed farther away from the anode as there are fewer opportunities for DEET in the outer regions of the biofilm. Over the past decade there has been a debate regarding the PEET mechanism in *G. sulfurreducens* and whether electrical conduction occurs via pili or redox-hopping through pilin-associated cytochromes. The latter was supported by confocal resonant Raman microscopy elucidating the spatial orientation and redox state of *c*-type cytochromes (Lebedev et al., 2014).

*S. oneidensis* was demonstrated to transfer electrons and generate electricity via SEET using reverse-phase liquid chromatography-MS coupled with secondary MS analysis and slow-scan voltammetry (Marsili et al., 2008). Simultaneous short circuit current measurements and optical imaging demonstrated a substantial current increase after the formation of cell/nanoparticle aggregates (Jiang et al., 2014). These aggregates serve as “bridges” to facilitate EET from *Shewanella* cells to anode surfaces and also between interconnected cell networks. Enhanced current output was due to improved EET at the cell-electrode interface and within the extended cellular networks.

Spectro-electrochemical techniques allow investigation of the structure and function of cytochromes. These methodologies can also be used to determine the EET mechanism operating within anodophilic biofilms *in vivo*.

## CONCLUDING REMARKS

The microbial diversity of BESs highlights the need for robust methods to understand population dynamics, cell–cell communication and molecule-surface interactions. There is a need to directly evaluate the metabolism of cells growing on electrodes *in situ* to better understand the EET process. Genetic manipulation of key genes involved in EET, via deletion and overexpression, and subsequent *in vivo* monitoring of the EET is required to determine the role of each protein in EET. Knowledge gained on the structure, localization and function of proteins involved in these EET pathways can be used to monitor the performance of large-scale mixed community BESs. This review highlighted the need to approach the study of electrode-associated biofilms using multiple techniques to allow a more in-depth understanding of what is occurring at the electrode surface. Although numerous techniques exist to monitor the real-time gene-expression profiles of BES communities, most have not been fully applied to mixed community analysis in large-scale reactors. Further developments of analytical techniques elucidating which organisms and their associated molecular structures are responsible for EET to electrodes will enable successful materialization of the powerful applications of BESs.

### Conflict of Interest Statement

The authors declare that the research was conducted in the absence of any commercial or financial relationships that could be construed as a potential conflict of interest.
